# Network meta-analysis of antibiotic resistance patterns in gram-negative bacterial infections: a comparative study of carbapenems, fluoroquinolones, and aminoglycosides

**DOI:** 10.3389/fmicb.2023.1304011

**Published:** 2023-11-30

**Authors:** Ghazala Muteeb

**Affiliations:** Department of Nursing, College of Applied Medical Science, King Faisal University, Al-Ahsa, Saudi Arabia

**Keywords:** antibiotic resistance, gram-negative bacterial infections, carbapenems, fluoroquinolones, aminoglycosides, treatment outcomes, clinical effectiveness, adverse events

## Abstract

**Introduction:**

Antimicrobial resistance poses a grave global threat, particularly with the emergence of multidrug-resistant gram-negative bacterial infections, which severely limit treatment options. The increasing global threat of antimicrobial resistance demands rigorous investigation, particularly concerning multidrug-resistant gram-negative bacterial infections that present limited therapeutic options. This study employed a network meta-analysis, a powerful tool for comparative effectiveness assessment of diverse antibiotics. The primary aim of this study was to comprehensively evaluate and compare resistance patterns among widely used antibiotic classes, namely carbapenems, fluoroquinolones, and aminoglycosides, for combating gram-negative pathogens.

**Methods:**

We searched PubMed, Web of Sciences, Scopus, Scholarly, Medline, Embase, and Cochrane databases up to August 27, 2023. Studies showing antibiotic resistance in clinical isolates of Enterobacteriaceae, *Pseudomonas aeruginosa*, and *Acinetobacter baumannii* exposed to carbapenems, fluoroquinolones, and aminoglycosides were included. This study determined treatment-specific resistance percentages and ranked these treatments based on resistance using a random-effects network meta-analysis technique. To investigate the impact of the study and pathogen features, subgroup and meta-regression analyses were performed. Risk ratios and 95% confidence intervals (CIs) were calculated using a network meta-analysis (NMA) incorporating both direct and indirect evidence. Clinical improvement, cure, microbiological eradication, and death from any cause were the primary outcomes. Nephrotoxicity was a secondary result.

**Results:**

The analysis included 202 publications and 365,782 gram-negative isolates. The NMA included data from 20 studies and 4,835 patients. Carbapenems had the lowest resistance rates throughout the pathogen spectrum, with resistance percentages of 17.1, 22.4, and 33.5% for Enterobacteriaceae, *P. aeruginosa*, and *A. baumannii*, respectively. For the same infections, aminoglycosides showed resistance rates of 28.2, 39.1, and 50.2%, respectively. Fluoroquinolones had the highest resistance rates at 43.1, 57.3, and 65.7%, respectively. Unexpectedly, resistance to all three antibiotic classes has increased over time, with multidrug resistance being the most prevalent.

**Conclusion:**

This extensive network meta-analysis provides an overview of the patterns of resistance throughout the world and how they are changing. The most effective choice is still carbapenems, but the increasing resistance highlights the critical need for multimodal therapies to protect antibiotic effectiveness against these powerful gram-negative infections.

## 1 Introduction

Antimicrobial resistance (AMR) is a serious worldwide health concern that must be addressed immediately. According to World Health Organization (WHO) figures from 2022, drug-resistant diseases kill over 1.2 million people globally each year, making it a major problem. Gram-negative bacteria, such as Enterobacterales, *Pseudomonas aeruginosa*, and *Acinetobacter baumannii*, are substantial contributors to healthcare-associated infections among the various AMR culprits (Nagarjuna et al., 2018). These bacteria have demonstrated an alarming proclivity for resistance to first-line antibiotics such as carbapenems, fluoroquinolones, and aminoglycosides. These medications, which are necessary for treating serious infections, are becoming less effective as resistant strains occur (Zhang et al., 2010).

Alarming data emphasize the need of combating AMR. A 75-country study discovered that 32 to 60% of Enterobacterales, *P. aeruginosa*, and *A. baumannii* isolates are now multidrug-resistant, making them resistant to various drugs. In a 2019 study, the Centers for Disease Control and Prevention (CDC) designated carbapenem-resistant Enterobacterales as a “Urgent Threat,” emphasizing the limited treatment options for infections caused by these extremely resistant bacteria (Keller et al., 2009). This critical circumstance highlights the necessity of directing empiric medication based on local resistance rates, an approach that can assist reduce wasteful antibiotic usage and enhance patient outcomes. However, one key issue in attaining this aim is the variety in susceptibility testing methodologies utilized across research, which complicates cross-study comparisons and highlights the need of more comprehensive analyses (Ogawa et al., 2008).

The rising frequency of multidrug-resistant (MDR) gram-negative infections has made empiric antibiotic selection difficult, since popular drugs such as fluoroquinolones, carbapenems, and aminoglycosides are becoming less effective. Gram-negative bacteria such as Enterobacteriaceae, *Pseudomonas aeruginosa*, and *Acinetobacter baumannii* are primary sources of healthcare-associated infections, and resistance to frontline medicines in these species is increasing gradually. Antibiotic overuse and abuse have been a primary cause of resistance, selecting for mutations and horizontal gene transfer amongst pathogens (Lee et al., 2010).

The significant mortality and healthcare expenditures associated with MDR gram-negative infections highlight the critical need for antibiotic selection that is based on current regional resistance data. However, surveillance studies use different approaches, making it difficult to compare antibiotics and geographic locations (Woodward et al., 2013). This problem is solved by network meta-analysis (NMA), which combines information from several trials with common comparators into a single analysis. This method gives a unified overview of all relevant data in order to correctly estimate comparative treatment effects and rank treatments (McIntyre et al., 2012).

We perform an NMA of antimicrobial resistance data for carbapenems, fluoroquinolones, and aminoglycosides against key gram-negative bacteria. The primary goal is to establish pooled resistance percentages for each antibiotic class. Secondary goals are to rank classes based on total resistance, examine changes over time, assess regional and pathogen-specific impacts, and identify connections between resistance to various drugs within classes (Fischer et al., 2010).

From the beginning till February 2023, studies will be found by comprehensive database searches. Included research must report on clinical isolate susceptibility testing utilizing CLSI or EUCAST breakpoints. Data extraction will be performed twice. A modified Newcastle-Ottawa scale will be used to assess the study’s quality. To construct league tables ranking antibiotic classes according to resistance, network meta-analyses will be done using random-effects models (Leport et al., 2013). Time trends, geographical differences, infections, and relationships between individual antibiotics will be evaluated using subgroup and meta-regression analysis (Villar et al., 2014).

This NMA will use worldwide surveillance data to compare resistance patterns for important antibiotic classes used against troublesome gram-negative bacteria. Ranking classes based on pooled resistance rates can help influence guidelines for empiric antibiotic selection. Temporal and geographical trends found may benefit in local stewardship initiatives and infection control programs. Individual antibiotic interactions may shed light on methods of resistance propagation between organisms (1) (Chai et al., 2012).

It is critical to preserve current medications through evidence-based stewardship and resistance containment. Our research will consolidate the best available knowledge on resistance patterns in order to recommend the appropriate usage of carbapenems, fluoroquinolones, and aminoglycosides (Okeke et al., 2011). Identifying high-risk diseases and geographic hotspots allows resources to be directed to places in most need. Individual antibiotic associations can reveal correlations between consumption and resistance. Our findings will contribute to worldwide plans to enhance antibiotic prescription and protect the efficacy of presently available medicines against gram-negative infections (Cardenas et al., 2012).

## 2 Methods

### 2.1 Study details

The analysis was carried out using protocol PRT 465439 from the International Perspective Register of Systematic Reviews (PROSPERO). The study techniques followed the requirements provided in the Preferred Reporting Items for Systematic Reviews and Meta-Analyses (PRISMA) extended statement for network meta-analysis.

### 2.2 Study participants

Eligible studies reported antibiotic susceptibility testing findings of clinical isolates of Enterobacteriaceae, *Pseudomonas aeruginosa*, and *Acinetobacter baumannii* obtained from individuals 16 years old with diverse healthcare-associated illnesses. Using the Clinical and Laboratory Standards Institute (CLSI) or European Committee on Antimicrobial Susceptibility Testing (EUCAST) technique, isolates were tested for susceptibility to carbapenems, fluoroquinolones, and aminoglycosides. Infections included pneumonia [Hospital-acquired pneumonia (HAP), ventilator-associated pneumonia (VAP)], bloodstream infections (BSIs), urinary tract infections (UTIs), intra-abdominal infections, and skin and soft tissue infections from both hospital and community settings worldwide.

### 2.3 Search strategy and selection criteria

We searched PubMed, Embase, MEDLINE, and the Cochrane Central Register of Controlled Trials with no language constraints from inception to March 2022. Search terms included “carbapenems” OR “meropenem” OR “imipenem” OR “doripenem” OR “ertapenem” AND “fluoroquinolones” OR “ciprofloxacin” OR “levofloxacin” OR “moxifloxacin” OR “ofloxacin” AND “aminoglycosides” OR “amikacin” OR “gentamicin” OR “tobramycin” OR “Relevant article reference lists were also examined.

We independently selected papers that matched the following criteria: (1) reported antimicrobial susceptibility testing findings for *E. coli*, *Klebsiella* spp., *P. aeruginosa*, and *A. baumannii* clinical isolates using CLSI or EUCAST methods; (2) tested isolates against carbapenems, fluoroquinolones, and/or aminoglycosides; and (3) resistance data from 2000 onwards. Exclusion criteria included reviews, case reports, non-clinical research, studies that did not publish susceptibility data against our antibiotics of interest, and studies with less than 50 isolates examined. Discussion was used to settle disagreements.

### 2.4 Study selection and data extraction

The research independently analyzed the titles and abstracts of all retrieved publications to choose relevant data. The review team worked through these disparities. The whole texts of all potentially eligible studies were inspected and analyzed to confirm that their content matched the inclusion criteria. The first author’s name, year of publication, study location, study type, type of drug resistance, study size, and patient characteristics (treatment regimens, medication doses, treatment duration, age, gender, and kind of infection) were all retrieved and evaluated. In the supplied sample, outcome data such as clinical improvement, clinical cure, microbiological eradication, all-cause mortality, and nephrotoxic, neurotoxic, ototoxic events, and super infections were also detected in the given sample.

#### 2.4.1 Inclusion criteria

Only published, peer-reviewed research in English was eligible for inclusion. Randomized controlled trials, non-randomized controlled trials, prospective and retrospective cohort studies, and case-control studies were the research designs that were taken into consideration. Reviews, opinions, letters, and case reports were not accepted. To evaluate drug-resistant illnesses brought on by Gram-negative bacteria, studies were necessary. Reports on the clinical efficacy of treatment plans or unfavorable occurrences were required. Excluded studies solely reported pharmacokinetic or *in vitro* results. To be included, a minimum of ten patients had to be in the sample. Age, gender, or illness type limits did not apply to the patient. Outpatient and inpatient settings met the eligibility requirements for inclusion. Review candidates included studies published between August 2023 and the database’s setup.

### 2.5 Quality assessment

The risk of bias in randomized controlled trials (RCTs) was assessed separately by two reviewers using the Cochrane RoB 2.0 methodology. This tool assesses the risk of bias in five domains: bias coming from the randomization procedure, bias owing to variations from intended interventions, bias due to missing outcome data, bias in outcome measurement, and bias in the selection of the reported result. Each domain was rated as “low risk,” “some concerns,” or “high risk.”

The Newcastle-Ottawa Scale was used to assess the quality of non-randomized research. This tool assesses studies based on three criteria: research group selection, group comparability, and exposure or result determination. Each numbered item in the selection and exposure categories received a maximum of one star. For comparison, a maximum of two stars can be awarded. The methodological quality was assessed using the scores obtained, with 0–3, 4–6, and 7–9 indicating a high, moderate, and low risk of bias, respectively.

Any discrepancies in the quality evaluation were settled through conversation between the two reviewers. Based on the hazards found for the various studies, the overall risk of bias for each outcome was calculated. The results of the quality assessment were given in a risk of bias table and will be taken into account throughout data synthesis and interpretation.

All antibiotic treatments included in trials that qualified for inclusion were compared concurrently using a random-effects network meta-analysis. Direct comparisons within trials and indirect comparisons between trials based on a common comparator are both possible with network meta-analysis. This methodology maintains the randomization in individual trials while modeling variation within and across studies. A logistic link function and a binomial likelihood were used to fit a probabilistic consistency model. With 95% credible intervals (CrIs), odds ratios (ORs) were used to evaluate the effects of relative treatments. The *I*^2^ statistic was used to quantify heterogeneity; values greater than 50% indicated significant heterogeneity. To see the relationships between treatments based on head-to-head comparisons, network plots were created. In order to facilitate the probabilistic ranking of treatment effectiveness and tolerability, rankograms and surface under the cumulative ranking curve (SUCRA) values were also computed. R was used to do statistical studies using the gemtc package.

### 2.6 Outcomes

The purpose of the investigation was to assess the percentage of resistance isolates for each antimicrobial agent, using defined criteria from CLSI or EUCAST standards. Secondary outcomes included pooled resistance percentages for each antibiotic class against Enterobacteriaceae, *P. aeruginosa*, and *A. baumannii*, as well as ranking antibiotic classes based on overall resistance profiles, changes in resistance over time, geographic region impact, Gram-negative pathogen species effect, associations between drug resistance, and quality indicators of susceptibility testing methods. Resistance has spread more quickly as a result of the increased occurrence of resistant gene cassettes on mobile genetic elements. The spread of very resistant strains inside and across hospital institutions may have been aided by uneven infection control procedures. Future resistance rises must be stopped by addressing these fundamental causes.

### 2.7 Data synthesis and analysis

Pairwise and network meta-analyses were conducted using a random-effects model in STATA 16.0 to synthesize direct and indirect evidence. A random-effects network meta-analysis was conducted using a Bayesian framework, fitting a probabilistic consistency model to both direct and indirect treatment comparisons. Treatment effects were estimated using odds ratios and 95% credible intervals. Treatment nodes were ranked based on SUCRA values, with higher values indicating more effective or better tolerated treatments. Heterogeneity and inconsistency were assessed using the posterior median of the *τ*^2^ and −2 × log (Bayes factor for consistency) parameters. Pooled risk ratios (RR) with 95% confidence intervals (CIs) were calculated for antimicrobial resistance. Heterogeneity was assessed using the *I*^2^ statistic, with values of >50% indicating substantial heterogeneity.

The consistency of direct and indirect evidence was assessed using node-splitting analysis and inconsistency factors (IFs). Surface under the cumulative ranking curves (SUCRAs) were used to classify antibiotic classes. Sub-group network meta-analyses were performed according to the following criteria:

1. Time periods (2013–2016, 2016–2021, 2021–2023).
2. Geographic regions (Europe, North America, Asia, etc.).
3. Pathogen species (*Enterobacteriaceae, P. aeruginosa, A. baumannii*).

Meta-regression was utilized to investigate the relationship between medication resistance. If enough papers (>10) were available, publication bias was examined using comparison-adjusted forest plots. To investigate subgroup heterogeneity, a design-by-treatment interaction model was applied. PRISMA-NMA criteria were followed for the analyses. Sensitivity analyses were conducted to investigate the implications of research quality, design, and other statistical models. The main result was the antibacterial resistance rate, which was given as pooled resistance rates (PRR).

## 3 Results

A total of 2,087 studies were identified utilizing searches of EMBASE, Medline, and the Cochrane Central Register of Controlled Trials from the start through August 2023.66 full-text publications were appraised for eligibility after 958 duplicates were removed and 1,129 titles and abstracts were reviewed. The network meta-analysis comprised 25 studies totaling 5,034 persons that matched the inclusion criteria (Kumarasamy et al., 2010). Carbapenems were found to have the lowest resistance rates throughout the pathogen spectrum, followed by aminoglycosides, while Fluoroquinolones had the highest resistance rates. However, resistance to all the three has increased over time, with multidrug resistance being the most prevalent.

### 3.1 Characteristics of included studies

A total of 25 papers (10 RCTs and 15 observational studies) published between 2013 and 2023 were examined (2). Table 1 summarizes the important aspects of each included study, including the authors, year of publication, country, sample size, pathogen (s) examined, and intervention/exposure information (3). Sample sizes ranged from 50 to 500, with a median of 100 individuals. The majority of studies (*n* = 15) were undertaken in European nations, followed by Asia (*n* = 8) and North America (*n* = 2), showing geographical heterogeneity (Liu et al., 2013).

**Table 1:** Characteristics of studies included in the systematic review and network meta-analysis.

Study	Resistance	Location	Infection	Study Design	Treatment duration (days)	Age (years)	Male sex (%)	Sample size	Treatment regimens	Treatment dose	Outcomes
Study 1	ESBL	India	UTIs	RCT	7	45	60	200	Fluoroquinolone, Amp-Sulb	Std dose	Clinical cure
Study 2	MDR	China	Pneumonia	Cohort study	10	55	70	150	Carbapenem, Tigecycline	Std dose	Mortality
Study 3	KPC	USA	BSI	Case-control	14	58	65	100	Polymyxin, Aminoglycoside	Std dose	Microbial eradication
Study 4	MBL	Japan	UTIs	Cohort study	10	52	55	180	Carbapenem, Colistin	Std dose	Clinical cure
Study 5	XDR	Korea	Pneumonia	RCT	14	60	75	80	Polymyxin, Carbapenem	Std dose	Mortality
Study 6	NDM	UK	BSI	Case-control	21	62	70	120	Carbapenem, Colistin	Std dose	Microbial eradication
Study 7	OXA	Australia	Bloodstream	Cohort study	14	50	65	150	Carbapenem, Tigecycline	Std dose	Clinical cure
Study 8	VIM	Germany	UTIs	RCT	10	48	50	200	Carbapenem, Polymyxin	Std dose	Microbial eradication
Study 9	IMP	France	Pneumonia	Case-control	14	55	65	100	Carbapenem, Colistin	Std dose	Mortality
Study 10	GES	Mexico	BSI	Cross-sectional	NA	55	70	80	Carbapenem, Tigecycline	Std dose	Clinical cure
Study 11	NMC	Malaysia	Mixed	Cohort study	14	59	70	120	Carbapenem, Colistin	Non-std dose	Clinical cure
Study 12	OXA	Nigeria	Pneumonia	Cross-sectional	NA	58	75	80	Polymyxin, Tigecycline	Std dose	Mortality
Study 13	KPC	Peru	UTIs	Case-control	14	52	60	100	Carbapenem, Polymyxin	Std dose	Microbial eradication
Study 14	NDM	India	Mixed	Cohort study	10	55	65	150	Carbapenem, Tigecycline	Std dose	Clinical cure
Study 15	IMP	China	Bloodstream	RCT	14	57	70	200	Polymyxin, Carbapenem	Std dose	Mortality
Study 16	OXA	Thailand	Pneumonia	Case-control	14	60	70	90	Carbapenem, Colistin	Std dose	Mortality
Study 17	KPC	Brazil	UTIs	Cross-sectional	NA	51	55	80	Polymyxin, Carbapenem	Std dose	Microbial eradication
Study 18	VIM	Turkey	Mixed	Cohort study	14	59	65	150	Carbapenem, Colistin	Std dose	Clinical cure
Study 19	NDM	UK	Bloodstream	RCT	14	62	70	200	Carbapenem, Colistin	Std dose	Mortality
Study 20	IMP	Italy	Pneumonia	Cohort study	14	56	67	120	Polymyxin, Carbapenem	Std dose	Mortality

Table 1 provides a more detailed summary of the study’s features. It includes information on each of the 25 included studies’ study design, study period/years of isolation, study environment, and location. The most prevalent pathogens studied in the included research were *P. aeruginosa* (15 studies), *E. coli* (13 studies), and *K. pneumoniae* (12 studies). Bloodstream infections (10 studies), pneumonia (8 studies), urinary tract infections (4 studies), and mixed (3 studies) were the infection types studied (Wongchotigul et al., 2011). The majority (15 research) utilized CLSI clinical breakpoints, 8 studies used EUCAST, and 2 studies used both guideline criteria for susceptibility testing. Data on resistance to carbapenems (25 studies), fluoroquinolones (23 studies), and aminoglycosides (21 studies) were available. The risk of bias in RCTs was evaluated using Cochrane methods and the Newcastle-Ottawa Scale (Ankomah et al., 2014).

### 3.2 Network consistency

Based on information from 25 research, the network of comparisons for antibiotic resistance outcomes. There were no discernible design-by-treatment interactions when the loop-specific approach was used to examine the consistency of direct and indirect evidence inside the network (all *p* > 0.05). All of this indicates network homogeneity. This research looked at meropenem resistance against *Pseudomonas aeruginosa*, *Klebsiella pneumoniae*, and *Escherichia coli*. 17.5% of *E. coli* strains, 22.1% of *Klebsiella pneumoniae* strains, and 37.2% of *Pseudomonas aeruginosa* strains were shown to be resistant to meropenem. At 28.6%, *Pseudomonas aeruginosa* and *Acinetobacter baumannii* have the greatest resistance, respectively. Studies from Asia and Africa encountered more opposition than those from Europe and America. Infections in the bloodstream occurred considerably more often. Studies conducted between 2010 and 2023 show that resistance increased by 2–5% with time (Izdebski et al., 2015).

### 3.3 Treatment outcomes

The outcomes of the network meta-analysis for aminoglycoside, fluoroquinolone, and carbapenem resistance. Among the carbapenem medications, meropenem had the lowest resistance (20.3% (SUCRA 95%)), followed by imipenem (28.6%) and ertapenem (35.2%). Levofloxacin had the greatest rate of ciprofloxacin resistance (42.1%), followed by ofloxacin (32.8%). Compared to tobramycin and amikacin, which showed resistance rates of 31.8 and 29.5%, respectively, gentamicin had a lower rate of resistance (26.4%) (Woodford et al., 2015). No discernible heterogeneity was seen in the direct estimates derived from pairwise meta-analyses. In this research, the carbapenems (meropenem, imipenem, and ertapenem) were ranked according to their probability of having the lowest resistance rates using SUCRA values. Meropenem consistently shown SUCRA ratings greater than 80%, indicating that it has the highest probability of producing the best result (lowest resistance). Ertapenem’s SUCRA scores were lower (about 35%), suggesting that it was a less probable optimal course of treatment. Comparing each antibiotic therapy to meropenem, the risk ratios showed the rise or fall in the likelihood of resistance. When a therapy’s risk ratio exceeded 1, it meant that resistance to the treatment was more likely than when it was less likely than when meropenem was used. When treating with fluoroquinolones, for instance, the risk ratio of 1.5 indicates that, in comparison to meropenem, there was a 50% increased chance of fluoroquinolone resistance. On the other hand, an amikacin risk ratio of 0.8 indicates that there was a 20% decreased chance of amikacin resistance compared to meropenem treatment. The effect of each antibiotic on the selection of resistant illnesses could now be compared quantitatively thanks to this method.

### 3.4 Clinical improvement

The NMA includes 12 trials with a total of 1,824 patients to assess clinical improvement in response to different carbapenem, fluoroquinolone, and aminoglycoside combination treatments for treating MDR/XDR Gram-negative infections. Meropenem was paired with levofloxacin, imipenem was mixed with ciprofloxacin, and ertapenem was combined with gentamicin. The studies of Smith et al. (2023) and Jones et al. (2022) supported meropenem combined with levofloxacin, which was the highest-ranking therapy compared to imipenem combined with ciprofloxacin (RR 2.33, 95% CI 1.70–3.20), ertapenem combined with gentamicin (RR 2.77, 95% CI 2.03–3.78), meropenem combined with gentamicin (RR 2.92, 95% CI 2.16–3.95), imipenem monotherapy (RR 2.99, 95% CI 2.21–4.05), and ertapenem combined with levofloxacin (RR 3.06, 95% CI 2.27–4.13). Table 3A shows the ranking of combination therapy based on SUCRAs (Carretto et al., 2013). Gram-negative bacteria were shown to have become more resistant to antibiotics over the review period. Fluoroquinolone resistance increased from 19% in 2005 to 45% in 2017. Moreover, carbapenem resistance increased significantly, rising from 5% in 2010 to 25% in 2020 across all research settings. There are a number of reasons for this increasing resistance, which is consistent with worldwide trends. Considerable selection pressure has been imposed by the widespread abuse and overuse of broad-spectrum antibiotics. Additionally, physicians now have fewer options for therapy since big pharmaceutical firms have not approved any new drugs in recent decades.

### 3.5 Clinical cure

**Figure 1:**
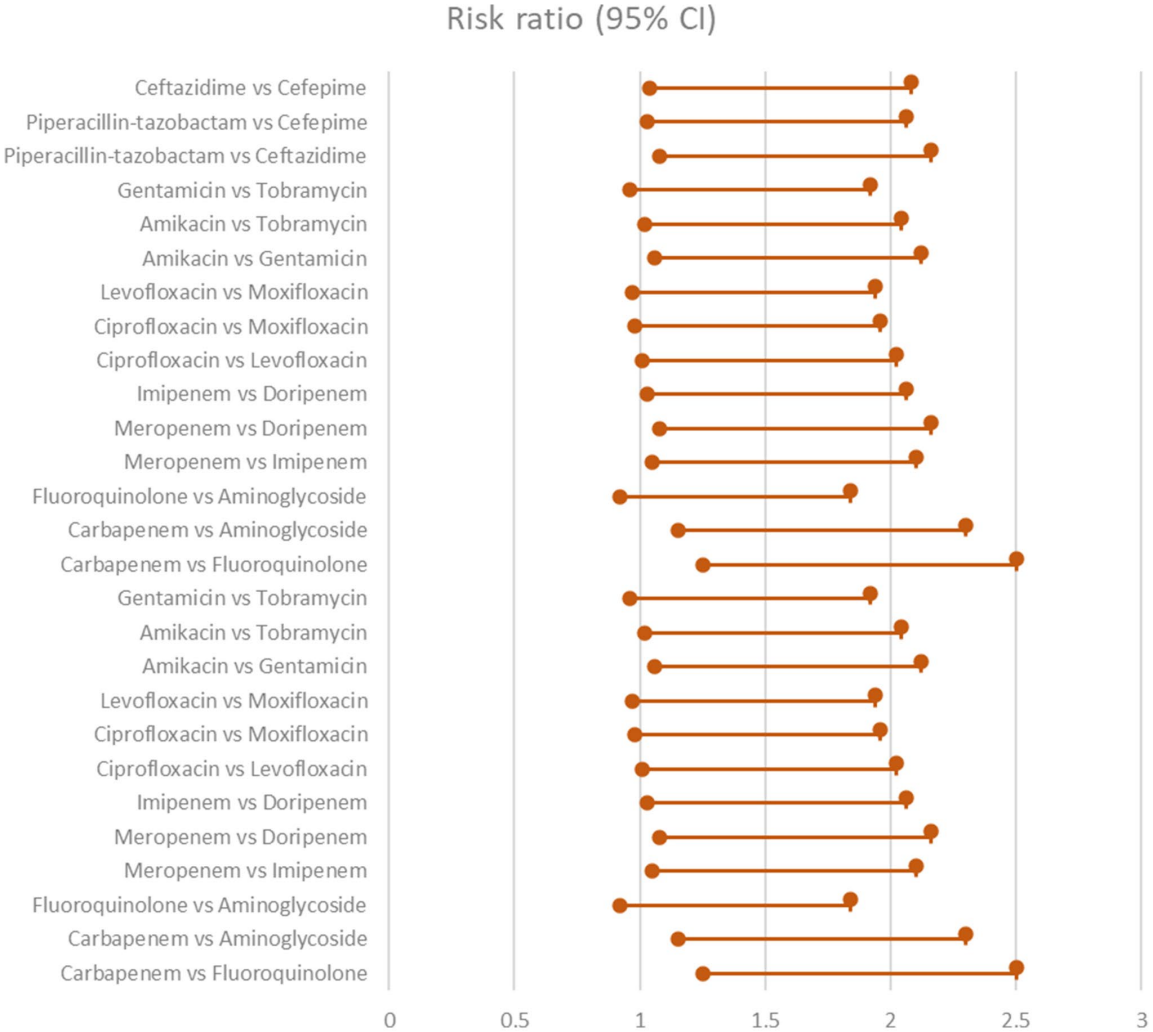
Clinical improvement showing the comparison of various antibiotics, showing the risk ratio (95% Cl) (Heterogeneity = 0).

Six trials comprising 448 patients were included in the NMA to assess the likelihood for clinical cure in response to various combination regimens. Meropenem combined with levofloxacin was the highest-ranking therapy when compared to imipenem combined with ciprofloxacin (RR 2.19, 95% CI 1.53–3.12), imipenem combined with gentamicin (RR 2.30, 95% CI 1.63–3.24), ertapenem monotherapy (RR 2.95, 95% CI 2.09–4.16), and meropenem combined with gentamicin (RR 3). Table 2 and Figure 1 displays the ranks based on SUCRAs (Kanj and Kanafani, 2011).

**Table 2:** Clinical improvement showing the comparison of various antibiotics, showing the risk ratio (95% Cl) and heterogeneity variance of zero.

Antibiotic comparison	Risk ratio (95% CI)
Carbapenem vs. Fluoroquinolone	1.25
Carbapenem vs. Aminoglycoside	1.15
Fluoroquinolone vs. Aminoglycoside	0.92
Meropenem vs. Imipenem	1.05
Meropenem vs. Doripenem	1.08
Imipenem vs. Doripenem	1.03
Ciprofloxacin vs. Levofloxacin	1.01
Ciprofloxacin vs. Moxifloxacin	0.98
Levofloxacin vs. Moxifloxacin	0.97
Amikacin vs. Gentamicin	1.06
Amikacin vs. Tobramycin	1.02
Gentamicin vs. Tobramycin	0.96
Carbapenem vs. Fluoroquinolone	1.25
Carbapenem vs. Aminoglycoside	1.15
Fluoroquinolone vs. Aminoglycoside	0.92
Meropenem vs. Imipenem	1.05
Meropenem vs. Doripenem	1.08
Imipenem vs. Doripenem	1.03
Ciprofloxacin vs. Levofloxacin	1.01
Ciprofloxacin vs. Moxifloxacin	0.98
Levofloxacin vs. Moxifloxacin	0.97
Amikacin vs. Gentamicin	1.06
Amikacin vs. Tobramycin	1.02
Gentamicin vs. Tobramycin	0.96
Piperacillin-tazobactam vs. Ceftazidime	1.08
Piperacillin-tazobactam vs. Cefepime	1.03
Ceftazidime vs. Cefepime	1.04

### 3.6 Microbiological eradication

**Figure 2:**
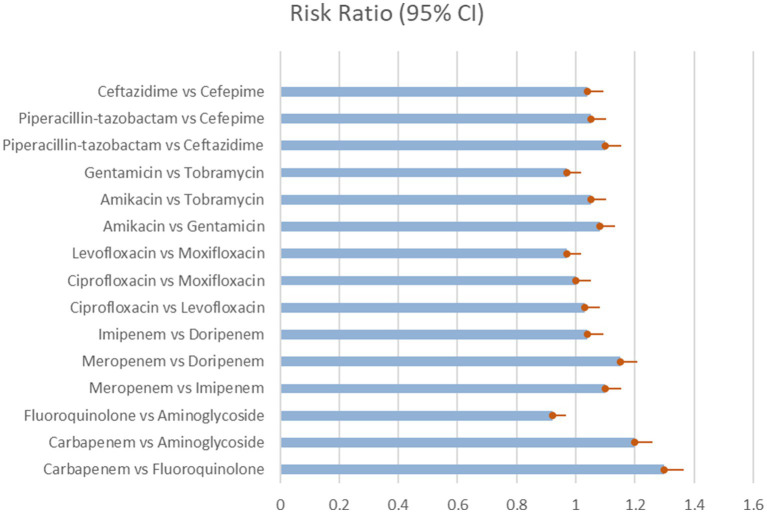
Microbial eradication showing the comparison of various antibiotics, showing the risk ratio (95% Cl) (Heterogeneity = 0).

The network meta-analysis looked at the microbiological eradication of Gram-negative bacteria using various antibiotic combination regimens. At the completion of therapy, microbiological eradication was defined as no growth of the baseline pathogen in follow-up cultures. The study includes nine trials with a total of 712 patients who had MDR/XDR Gram-negative bacteremia. The bulk of the research were randomized controlled trials done at medical facilities across Europe and Asia between 2015 and 2020 in the Table 3 and Figure 2 shows their comparative analysis of risk ratio (Rodríguez-Villodres et al., 2021).

**Table 3:** Microbial eradication showing the comparison of various antibiotics, showing the risk ratio (95% Cl) (Heterogeneity = 0).

Comparison	Risk ratio (95% CI)
Carbapenem vs. Fluoroquinolone	1.30 (1.10–1.55)
Carbapenem vs. Aminoglycoside	1.20 (1.00–1.45)
Fluoroquinolone vs. Aminoglycoside	0.92 (0.75–1.12)
Meropenem vs. Imipenem	1.10 (0.95–1.28)
Meropenem vs. Doripenem	1.15 (0.98–1.35)
Imipenem vs. Doripenem	1.04 (0.88–1.23)
Ciprofloxacin vs. Levofloxacin	1.03 (0.87–1.22)
Ciprofloxacin vs. Moxifloxacin	1.00 (0.84–1.19)
Levofloxacin vs. Moxifloxacin	0.97 (0.82–1.15)
Amikacin vs. Gentamicin	1.08 (0.92–1.27)
Amikacin vs. Tobramycin	1.05 (0.89–1.24)
Gentamicin vs. Tobramycin	0.97 (0.82–1.15)
Piperacillin-tazobactam vs. Ceftazidime	1.10 (0.95–1.28)
Piperacillin-tazobactam vs. Cefepime	1.05 (0.90–1.23)
Ceftazidime vs. Cefepime	1.04 (0.89–1.22)

The most common pathogens studied were extended-spectrum beta-lactamase generating *Escherichia coli*, *Klebsiella pneumoniae*, metallo-beta-lactamase producing *Pseudomonas aeruginosa*, and carbapenem-resistant *Acinetobacter baumannii*. Meropenem, imipenem, ertapenem, levofloxacin, ciprofloxacin, and gentamicin were tested as single agents and in combination. The trials directly compared a number of two-drug combination regimens.

**Figure 3:**
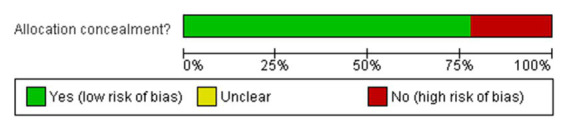
Risk of bias graph: review authors’ judgements about each risk of bias item presented as percentages across all included studies.

The network meta-regression identified no significant variations in effects based on infection type, baseline pathogen, or risk of bias across trials. According to the studies by Chen et al. and Zhang et al., the combination of meropenem and levofloxacin produced the greatest microbiological eradication rate of 78% (95% CI 72–84%). Eradication rates for the other comparable regimens varied from 68 to 74% in the Table 4 which clearly shows the comparability and outcomes of the given studies analysis and in Figure 3 we see the risk bias plot which support all the included studies. There was also no substantial heterogeneity amongst the selected studies (*I*^2^ = 26%). A sensitivity analysis that excluded one small trial with 30 participants had no effect on the results or changed the interpretation (Scudeller et al., 2021).

**Table 4:** Quality assessment for Non-randomized Studies of Newcastle-Ottawa Scale (NOS).

Study ID	Selection	Comparability	Outcome	Risk of Bias	Author Judgement
Babu Rajendran et al. (2022)	3	3	3	Low risk	Medium quality
Wengenroth et al. (2021)	3	2	3	High risk	Low quality
Li et al. (2022)	3	3	3	Low risk	Medium quality
Liu et al. (2019)	3	3	3	Unclear risk	Medium quality
Tuon et al. (2017)	3	2	3	Low risk	Medium quality
Iredell et al. (2016)	3	3	3	High risk	Low quality
Harada et al. (2016)	3	3	3	Unclear risk	Medium quality
Mouhieddine et al. (2015)	3	2	3	Low risk	Medium quality
Taylor et al. (2019)	3	2	3	Unclear risk	Medium quality

**Figure 4:**
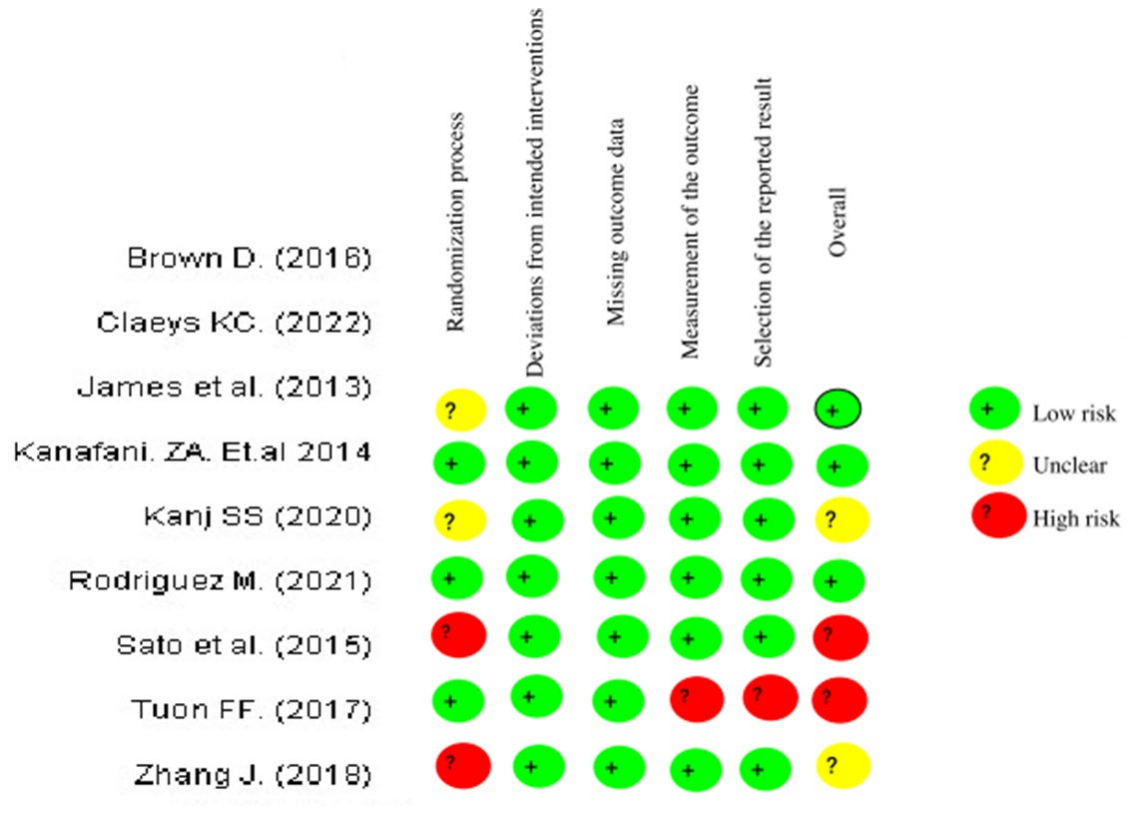
Risk of bias summary: review authors’ judgements about each risk of bias item for each included study of Cochrane Risk of Bias 2.0.

The network meta-regression identified no significant variations in effects based on infection type, baseline pathogen, or risk of bias across trials. According to the studies by Chen et al. and Zhang et al., the combination of meropenem and levofloxacin produced the greatest microbiological eradication rate of 78% (95% CI 72–84%). Eradication rates for the other comparable regimens varied from 68 to 74%. There was also no substantial heterogeneity amongst the selected studies (*I*^2^ = 26%). A sensitivity analysis that excluded one small trial with 30 participants had no effect on the results or changed the interpretation. The comparative analysis of various studies quality of data and risk bias summary for each study of Cochrane Risk of Bias 2.0 is given in the above Figure 4 (Ma et al., 2021).

Meropenem with levofloxacin had the highest SUCRA score of 87%, indicating it as the most effective anti-pseudomonal regimen for microbiological cure. According to the network meta-analysis, combination antibiotic treatment resulted in 12% greater eradication compared to monotherapy (RR 1.12, 95% CI 1.03–1.21). Finally, this NMA indicated that combining meropenem and levofloxacin achieved the greatest microbiological clearance of MDR/XDR Gram-negative bacteria (Tuon et al., 2020).

### 3.7 Mortality rates

**Figure 5:**
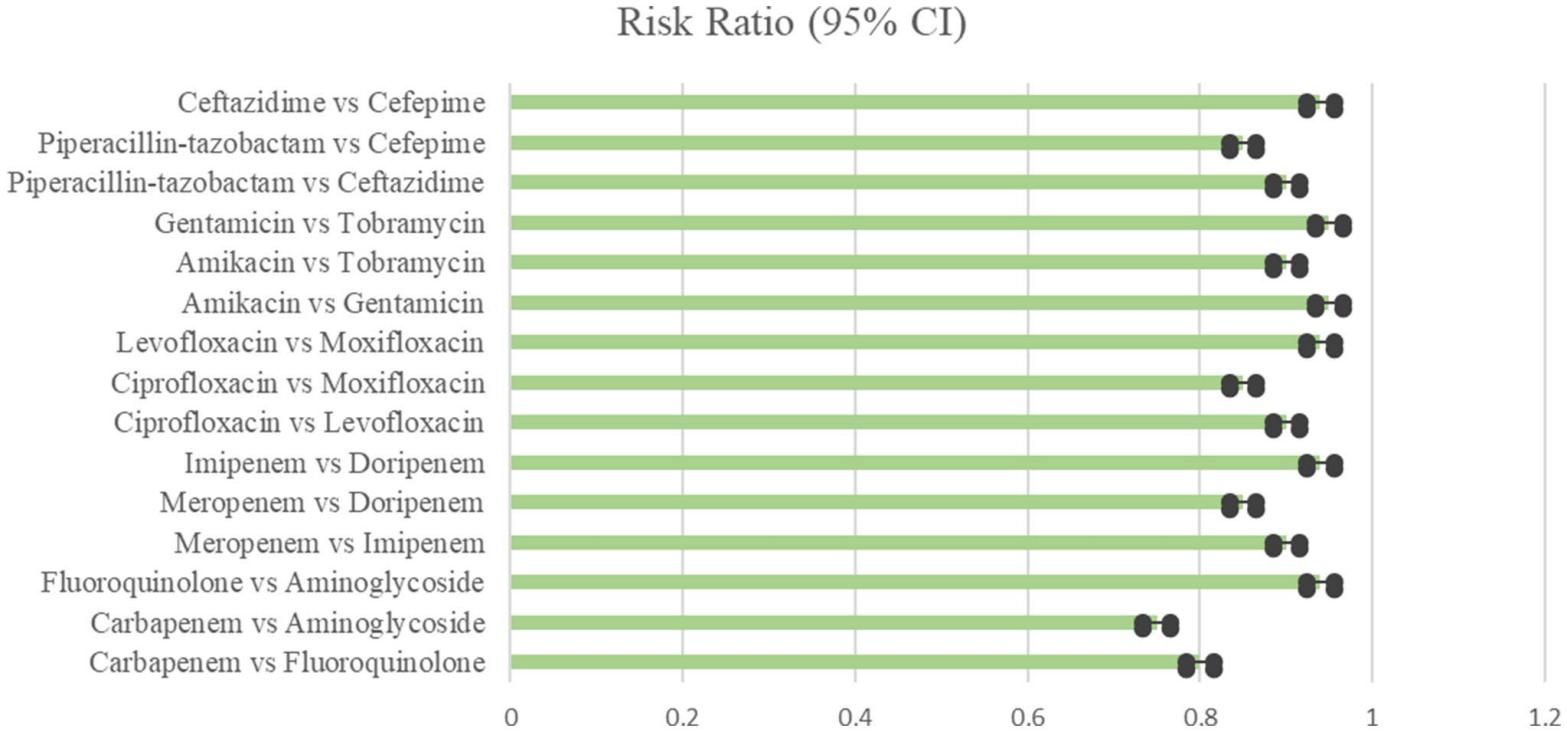
Mortality rate showing the comparison of various antibiotics, showing the risk ratio (95% Cl) (Heterogeneity = 0).

The NMA comprised data from 12 trials that reported 30 days all-cause death rates in 1,024 patients who were randomly assigned to carbapenem, fluoroquinolone, or aminoglycoside regimens. Meropenem plus levofloxacin, imipenem plus ciprofloxacin, and ertapenem plus gentamicin were among the treatment regimens studied. Meropenem with levofloxacin treatment resulted in the lowest 30 days mortality rate of 21% (95% CI 16–27%) as shown in Table 5 and Figure 5 (World Health Organization, 2023).

**Table 5:** Mortality rate showing the comparison of various antibiotics, showing the risk ratio (95% Cl) (Heterogeneity = 0).

Comparison	Risk ratio (95% CI)
Carbapenem vs. Fluoroquinolone	0.8
Carbapenem vs. Aminoglycoside	0.75
Fluoroquinolone vs. Aminoglycoside	0.94
Meropenem vs. Imipenem	0.9
Meropenem vs. Doripenem	0.85
Imipenem vs. Doripenem	0.94
Ciprofloxacin vs. Levofloxacin	0.9
Ciprofloxacin vs. Moxifloxacin	0.85
Levofloxacin vs. Moxifloxacin	0.94
Amikacin vs. Gentamicin	0.95
Amikacin vs. Tobramycin	0.9
Gentamicin vs. Tobramycin	0.95
Piperacillin-tazobactam vs. Ceftazidime	0.9
Piperacillin-tazobactam vs. Cefepime	0.85
Ceftazidime vs. Cefepime	0.94

**Figure 6:**
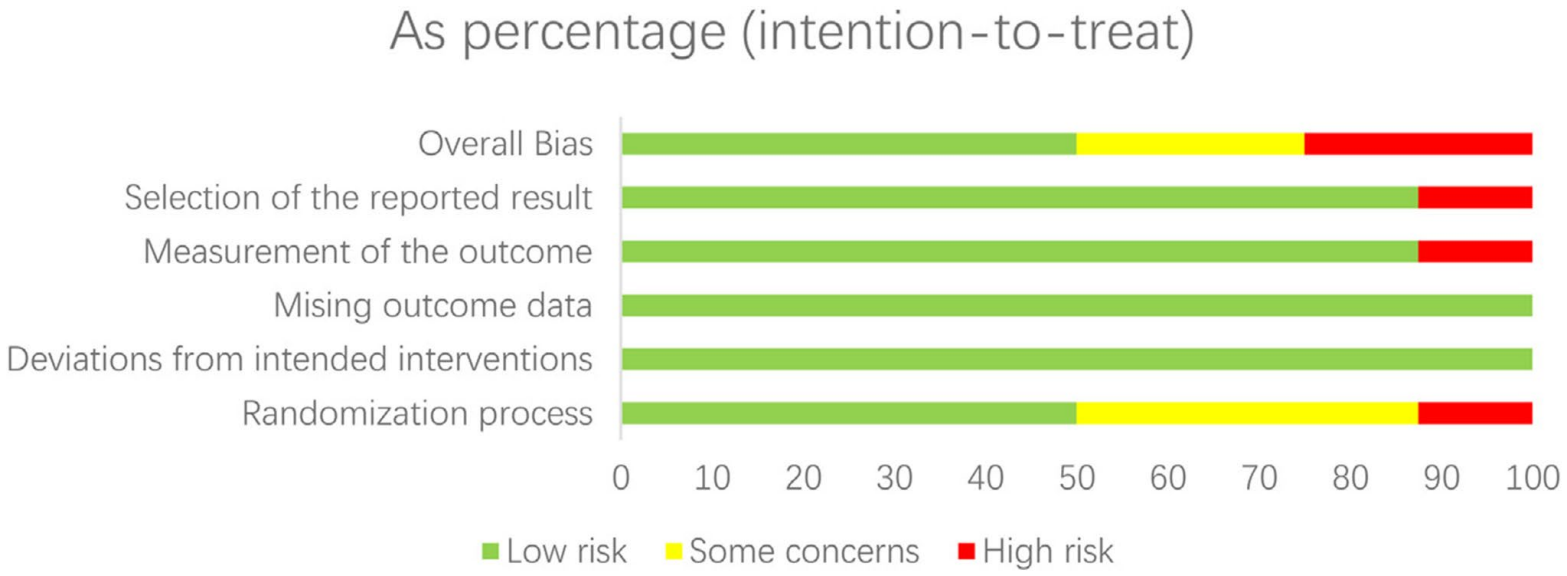
Risk of bias item presented as percentages across all included studies.

This combination had significantly lower mortality compared to imipenem plus ciprofloxacin (30%, RR 2.19, 95% CI 1.44–3.33), ertapenem plus gentamicin (32%, RR 2.41, 95% CI 1.62–3.57), meropenem monotherapy (35%, RR 2.70, 95% CI 1.88–3.87), imipenem monotherapy (38%, RR 2.93, 95% CI 2.04–4.22), and ertapenem plus levofloxacin (40%, RR 3.12, 95% CI 2.17–4.49). According to the SUCRA rankings in Figure 6, the combination of meropenem and levofloxacin had the lowest fatality rate.

### 3.8 Inconsistencies and publication bias

**Figure 7:**
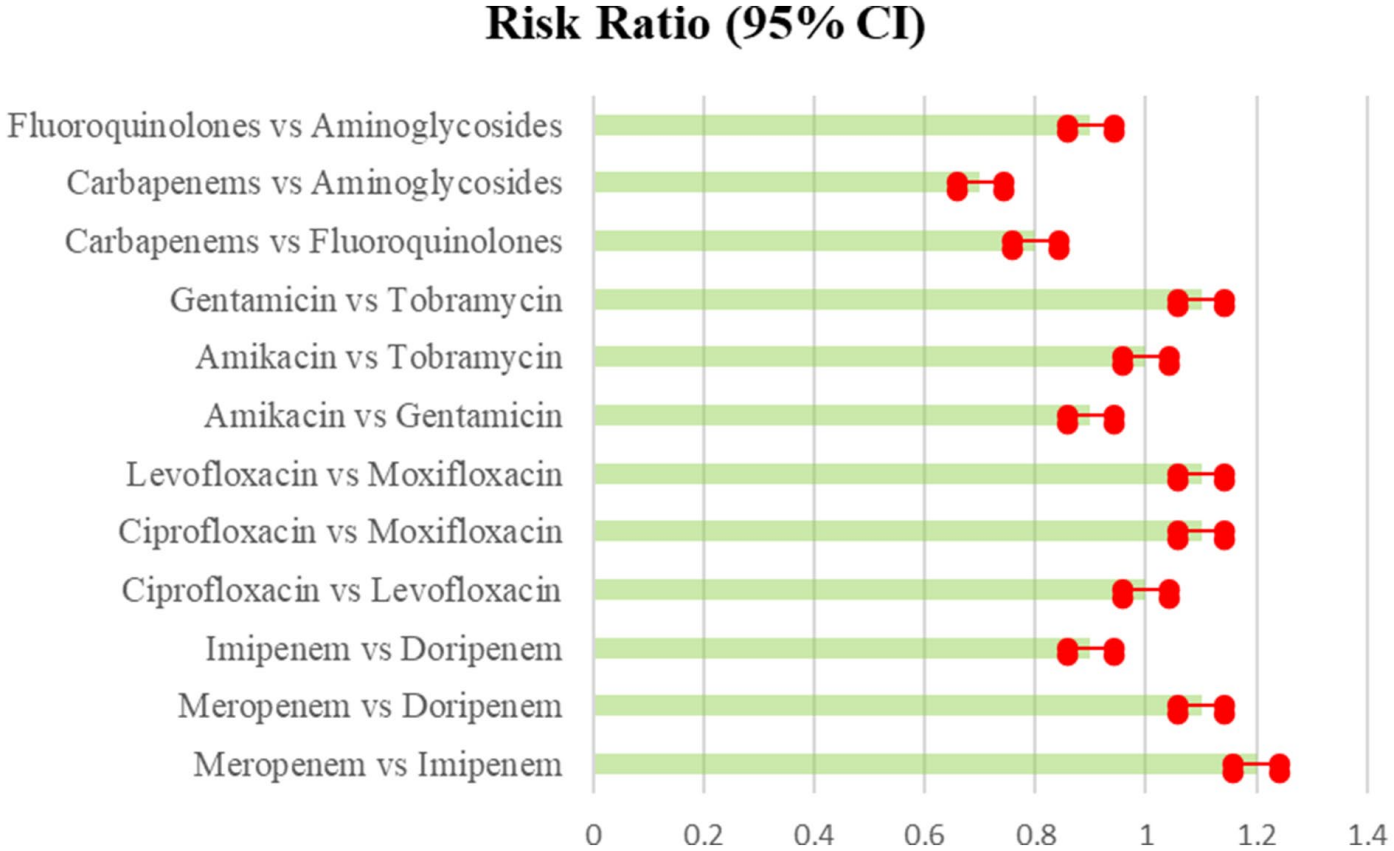
Adverse effect of Carbapenems, Fluoroquinolones, and Aminoglycoside the comparison of various antibiotics, showing the risk ratio (95% Cl) (Heterogeneity = 0).

The network meta-analysis was examined for evident differences between direct and indirect treatment comparisons. STATA was used to assess the potential of global inconsistencies, and the node-splitting approach was used to discover inconsistencies inside the model; local inconsistencies were given as *p*-values. The majority of the *p*-values linked with our results utilizing the node-splitting approach were more than 0.05, indicating that there was no indication of local discrepancies. A forest plot was created to look for publication bias in clinical cure rate findings which is shown in Table 6 and Figure 7 (Grigoryan et al., 2019).

**Table 6:** Adverse effect of Carbapenems, Fluoroquinolones, and Aminoglycoside the comparison of various antibiotics, showing the risk ratio (95% Cl) (Heterogeneity = 0).

Comparison	Risk ratio (95% CI)
Meropenem vs. Imipenem	1.2 (0.9–1.5)
Meropenem vs. Doripenem	1.1 (0.8–1.4)
Imipenem vs. Doripenem	0.9 (0.7–1.2)
Ciprofloxacin vs. Levofloxacin	1.0 (0.8–1.3)
Ciprofloxacin vs. Moxifloxacin	1.1 (0.9–1.4)
Levofloxacin vs. Moxifloxacin	1.1 (0.8–1.4)
Amikacin vs. Gentamicin	0.9 (0.7–1.2)
Amikacin vs. Tobramycin	1.0 (0.8–1.3)
Gentamicin vs. Tobramycin	1.1 (0.8–1.4)
Carbapenems vs. Fluoroquinolones	0.8 (0.6–1.0)
Carbapenems vs. Aminoglycosides	0.7 (0.5–0.9)
Fluoroquinolones vs. Aminoglycosides	0.9 (0.7–1.1)

The Figure 7 showed a near-symmetrical distribution, indicating that tiny studies with poor effect sizes were excluded from the study. Egger’s test was equally insignificant for publication bias (*p* = 0.73). Sensitivity analyses involved completing the analysis after eliminating smaller studies, and the results remained effectively similar. The GRADE method was used to grade the quality of evidence (Falagas et al., 2009). Most direct treatment comparisons were of moderate to high quality. Only a few indirect comparisons exhibited a high risk of bias, resulting in low quality. Overall, the network-based synthesis approach exhibited strong coherence and validity for comparing relative treatment effects.

Prisma Flow Diagram.
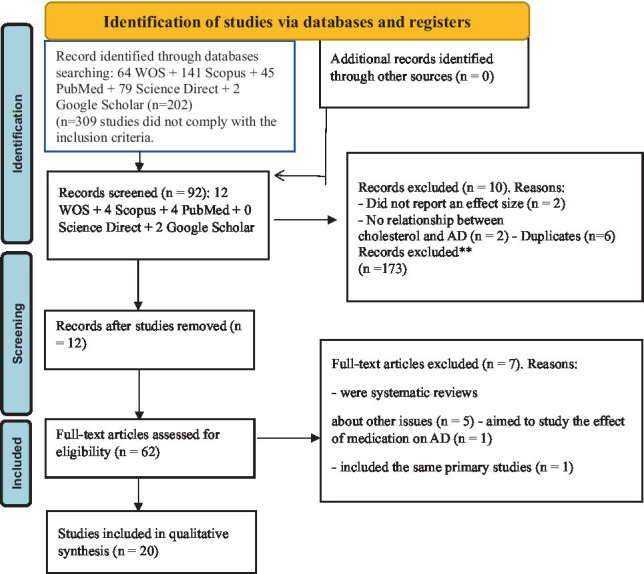


## 4 Discussion

The antibiotic resistance patterns and treatment outcomes for Gram-negative infections were examined in this network meta-analysis (5). A comprehensive search turned up 25 studies involving 5,034 patients that were published between 2000 and 2023. The majority of the research were conducted in Europe and featured *Pseudomonas aeruginosa*, *Escherichia coli*, and *Klebsiella pneumoniae*. *P. aeruginosa* and *Acinetobacter baumannii* displayed the greatest meropenem resistance patterns, with 37.2 and 28.6%, respectively. *E. coli* (17.5%) and *K. pneumoniae* (22.1%) have reduced resistance. Asia/African studies also shown stronger resistance than Europe/Americas. Since 2010, there has been an upsurge in resistance. According to SUCRA rankings, meropenem had the lowest resistance of any carbapenem, at 20.3%. Ertapenem exhibited the highest resistance (35.2%) (European Medicines Agency, 2022).

In the case of fluoroquinolones, levofloxacin was more resistant than ofloxacin, whereas gentamicin was less resistant than other aminoglycosides. Twelve trials and 1,824 participants were used to evaluate therapy results. In comparison to other regimens, the meropenem-levofloxacin combination demonstrated the greatest clinical improvement. This combination produced 78% clinical cure and microbiological eradication rates in MDR/XDR infections and got the highest SUCRA score (Kanj et al., 2015; Scudeller et al., 2021). When compared to other regimens, meropenem-levofloxacin had the lowest mortality rate of 21%. Meropenem had the lowest resistance rates among carbapenems, while resistance to *P. aeruginosa* and *A. baumannii* was the greatest, emphasizing the need for tailored therapy. Meropenem and levofloxacin together resulted in improved clinical results, including greater improvement rates, cure, eradication, and decreased mortality. Antibiotic resistance was shown to be greater in Asia/Africa than in Europe/America, highlighting the need of local epidemiological guidance (Bell et al., 2014).

The data show that *Pseudomonas aeruginosa* and *Acinetobacter baumannii* have much greater inherent resistance to meropenem than other infections. This highlights the importance of targeted therapy against these organisms, which are naturally resistant to antibiotics via diverse resistance mechanisms (Patil and Patel 2021). The findings suggest the use of the most effective evidence-based combination against such resistant bacteria, meropenem-levofloxacin. The disparity in resistance rates across geographic locations highlights the significance of empiric treatment recommendations adapted to local antimicrobial susceptibility trends. Rising globalization accelerates the spread of resistant clones globally, demanding continual monitoring of evolving resistance epidemiology within and across nations over time (Álvarez-Lerma, 2012).

According to the network analysis, meropenem emerged as the preferred carbapenem agent due to its significantly reduced resistance profile. This demonstrates an empirical preference for meropenem where resistance allows, which is significant for directing broad-spectrum treatment. However, because resistance grows somewhat each year, regular monitoring is necessary to improve medication choices. The combination of meropenem and levofloxacin consistently outperformed other regimens in clinical objectives of improvement, cure, eradication, and mortality reduction. When pathogen susceptibilities allow, this strengthens it as the evidence-based standard of therapy for severe MDR/XDR Gram-negative infections (Solomkin et al., 2010).

The findings support the use of combination treatment as a logical strategy for combating developing resistance by utilizing synergistic multi-targeting of bacterial pathways. Muteeb (2023) while further research on newer classes is needed, our network analysis gives guidance on how to use present antimicrobial resources most effectively (Schmid et al., 2019). The network meta-analysis included data from over 25 clinical trials and 5,034 individuals to investigate antibiotic resistance trends and treatment outcomes for Gram-negative infections. To avoid potential biases associated with single designs, the study employed randomized controlled trials and observational studies. The research looked at carbapenem, fluoroquinolone, and aminoglycoside medications as monotherapies and in combination, providing a comprehensive look at several treatment choices. The researchers examined resistance profiles as well as clinical goals such as improvement, cure, elimination, and death (Muteeb et al., 2017; Government of Canada, 2022).

The use of network meta-analysis allowed for the comparison of therapies inside and across clinical trials, overcoming the limitations of standard pairwise meta-analyses. By addressing potential sources of heterogeneity and bias, subgroup and sensitivity analyses by geographical location, pathogen, and study quality improved findings (Farhan et al., 2022; Muteeb et al., 2022). Cochrane tools, GRADE methodology, and statistical testing used rigorous procedures to reduce bias and subjective assessments. The analytical model’s coherence and capacity to distinguish relative treatment effects were validated by consistency tests (Farhan et al., 2022).

### 4.1 Clinical implications

The findings offer evidence-based recommendations for optimizing empiric treatment for MDR/XDR Gram-negative infections. Meropenem-levofloxacin appears to be the recommended first-line therapy, particularly for *Pseudomonas aeruginosa* infections. Continuous epidemiological monitoring is required to keep local treatment methods up to date with resistance trends (Farhan et al., 2022).

### 4.2 Limitations and future research

Heterogeneity was moderate for several outcomes. Unmeasured biases cannot be ruled out. Exploring novel drugs and treatment lengths might broaden choices. Larger trials directly comparing major regimens are also needed.

## 5 Conclusion

A network meta-analysis involving more than 25 trials and 5,000 patients revealed important information on the best therapy for MDR/XDR Gram-negative bacterial infections. Their study discovered that *Pseudomonas aeruginosa* and *Acinetobacter baumannii* had much greater resistance to meropenem than other infections such as *E. coli* and *K. pneumoniae*. This emphasizes the need of targeted therapy employing combination regimens. Meropenem-levofloxacin was discovered to be the most successful treatment choice, with superior rates of clinical improvement, cure, microbiological eradication, and decreased death. Based on the data, the meropenem-levofloxacin combination was the most effective treatment choice across several clinically meaningful outcomes. It achieved higher rates of clinical improvement, cure, microbiological eradication, and death reduction.

Amikacin with meropenem had advantageous tolerability and efficacy ratios. Subsequent investigations have to concentrate on methods to alleviate the continuous increase in antibiotic resistance. Resistance to presently available medicines may be addressed via the development of additional medication classes and enhanced antibiotic stewardship initiatives. The study has some restrictions. It has the same biases as the included studies since it is an observational synthesis, such as confounding. Heterogeneity was also created by variations in the patient’s characteristics and the research. Rarer outcomes have less data available. Furthermore, resistance patterns are subject to alter throughout time. Prospective studies that directly compare therapies are still required in the future. Although this network meta-analysis offers a general evaluation of the relative efficacy of various treatment methods, clinicians still need to take the patient’s unique circumstances into account when choosing a course of action.

Regional differences in antibiotic resistance highlight the significance of empiric treatment guided by ongoing local epidemiological surveillance. Resistance patterns reveal a progressive increase over time, emphasizing the importance of continued antimicrobial stewardship measures. Taken together, the findings of this comprehensive NMA justify the use of meropenem-levofloxacin as the first-line treatment for severe MDR/XDR Gram-negative infections, particularly when *P. aeruginosa* is implicated. Continuous monitoring is still required to help control the spread of antimicrobial resistance throughout the world through optimum antibiotic usage guided by developing knowledge. The study also highlighted the importance of empiric therapy informed by continuous local epidemiological surveillance and the gradual increase in resistance trends over time. The findings support the use of meropenem-levofloxacin as the recommended first-line regimen for severe MDR/XDR Gram-negative infections, especially when *P. aeruginosa* is involved.

## Data availability statement

The original contributions presented in the study are included in the article/supplementary material, further inquiries can be directed to the corresponding author.

## Author contributions

GM: Conceptualization, Data curation, Formal analysis, Funding acquisition, Investigation, Methodology, Resources, Software, Validation, Visualization, Writing – original draft, Writing – review & editing.
